# Quinazolinones as Competitive Inhibitors of Carbonic Anhydrase-II (Human and Bovine): Synthesis, *in-vitro, in-silico*, Selectivity, and Kinetics Studies

**DOI:** 10.3389/fchem.2020.598095

**Published:** 2020-12-01

**Authors:** Ajmal Khan, Majid Khan, Sobia Ahsan Halim, Zulfiqar Ali Khan, Zahid Shafiq, Ahmed Al-Harrasi

**Affiliations:** ^1^Natural and Medical Sciences Research Center, University of Nizwa, Nizwa, Oman; ^2^International Center for Chemical and Biological Sciences, H. E. J. Research Institute of Chemistry, University of Karachi, Karachi, Pakistan; ^3^Department of Chemistry, Government College University, Faisalabad, Pakistan; ^4^Institute of Chemical Sciences, Bahauddin Zakariya University, Multan, Pakistan

**Keywords:** quinazolinones, bovine carbonic anhydrase-II, human carbonic anhydrase-II, structure-activity relationship, kinetics, molecular docking

## Abstract

Carbonic anhydrase-II (CA-II) is associated with glaucoma, malignant brain tumors, and renal, gastric, and pancreatic carcinomas and is mainly involved in the regulation of the bicarbonate concentration in the eyes. CA-II inhibitors can be used to reduce the intraocular pressure usually associated with glaucoma. In search of potent CA-II inhibitors, a series of quinazolinones derivatives (**4a-p**) were synthesized and characterized by IR and NMR spectroscopy. The inhibitory potential of all the compounds was evaluated against bovine carbonic anhydrase-II (*b*CA-II) and human carbonic anhydrase-II (*h*CA-II), and compounds displayed moderate to significant inhibition with IC_50_ values of 8.9–67.3 and 14.0–59.6 μM, respectively. A preliminary structure-activity relationship suggested that the presence of a nitro group on the phenyl ring at R position contributes significantly to the overall activity. Kinetics studies of the most active inhibitor, **4d**, against both *b*CA-II and *h*CA-II were performed to investigate the mode of inhibition and to determine the inhibition constants (Ki). According to the kinetics results, **4d** is a competitive inhibitor of *b*CA-II and *h*CA-II with Ki values of 13.0 ± 0.013 and 14.25 ± 0.017 μM, respectively. However, the selectivity index reflects that the compounds **4g** and **4o** are more selective for *h*CA-II. The binding mode of these compounds within the active sites of *b*CA-II and *h*CA-II was investigated by structure-based molecular docking. The docking results are in complete agreement with the experimental findings.

## Introduction

Carbonic anhydrases (CAs, EC 4.2.1.1) are zinc-containing metallo-enzymes, found in animals, plants, algae, archaea, and eubacteria. CAs are encoded by three gene families, α-CA, β-CA, and γ-CA, that are evolutionarily unrelated (Hewett-Emmett, 2000; Jakubowski et al., 2018). These metallo-enzymes use zinc as a cofactor for the reversible inter-conversion of carbon dioxide and bicarbonate, while α-CAs possess high versatility, being able to catalyze other hydrolytic processes (Hewett-Emmett, 2000). Carbonic anhydrases are a class of hydrolase enzymes (Pocker and Meany, 1967; Arslan, 2001). In humans, more than 16 isoforms of carbonic anhydrase (*h*CA) are present (Shank et al., 2005; Shaik et al., 2019). CAs are involved in different physiological and pathological processes (Lindskog, 1997; Aggarwal et al., 2013; Ozensoy Guler et al., 2016). Consequently, these enzymes are interesting therapeutic targets for the treatment of pathological disorders (Chegwidden et al., 2000; Krishnamurthy et al., 2008; Supuran, 2008). CA-II is mainly involved in the regulation of the bicarbonate concentration in the eyes. CA-II inhibitors can be used to reduce the intraocular pressure usually associated with glaucoma (Supuran and Scozzafava, 2007; Pastorekova and Supuran, 2014; Ruusuvuori and Kaila, 2014; Zaraei et al., 2019). Moreover, CA-II is also expressed in malignant brain tumors (Parkkila et al., 1995) and renal, gastritis, and pancreatic carcinomas (Frazier et al., 1990; Pastorekova et al., 1997; Parkkila et al., 2000). The inhibitors of CA-II have also been considered as an adjunct in cancer chemotherapy (Zaraei et al., 2019).

These highly abundant proteins are involved in crucial physiological processes related with respiration. These enzymes are mainly involved in pH/CO_2_ homeostasis, secretion of electrolytes in tissues/organs, and transportation of CO_2_ and bicarbonate between the lungs and metabolizing tissues. Other than that these enzymes are also involved in many other physiological or pathological processes, such as bone resorption, gluconeogenesis, calcification, lipogenesis and ureagenesis, and tumorgenicity (Hewett-Emmett, 2000). CA-II has also been involved in glaucoma, epilepsy, leukemia, and cystic fibrosis (Achal and Pan, 2011; Sentürk et al., 2011).

**Figure 1:**
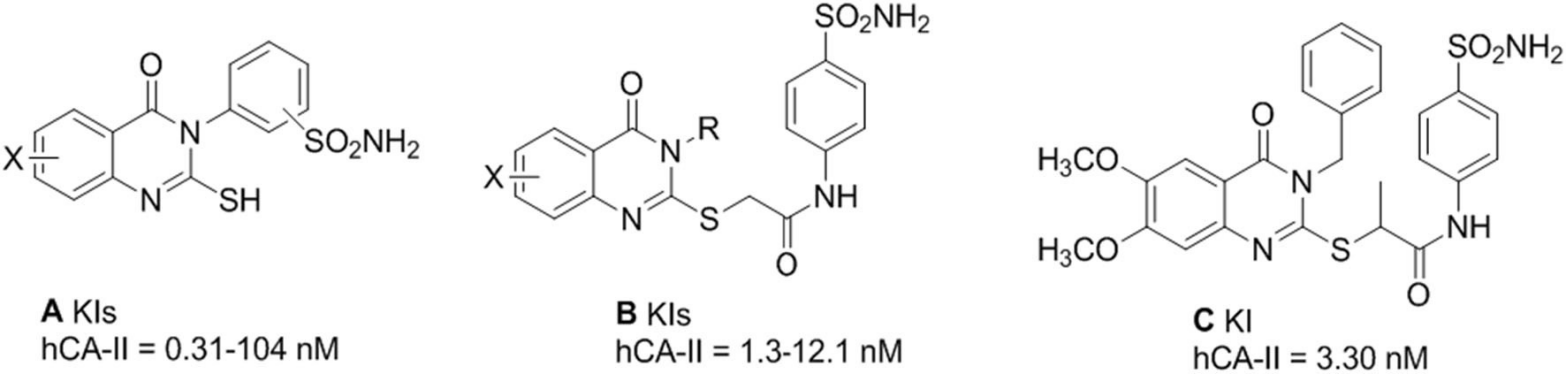
Reported inhibitors of CA-II **(A–C)**.

Quinazolinones are *N*-containing heterocyclic compounds that are widely distributed in nature, including in plants and microorganisms (He et al., 2017). Quinazolinone emerged as a privileged class of heterocyclic compounds with an increasing number of drug candidates and is regularly used in medicinal chemistry (Khan et al., 2014, 2015, 2016). The 2,3-Disubstituted quinazolinones retain anticancer (Al-Suwaidan et al., 2013), anticonvulsant (Gawad et al., 2011), anti-microbial (Al-Amiery et al., 2014), and anti-inflammatory activities (Alaa et al., 2016). Several derivatives of 3-(4-Aminosulfonyl)-phenyl-2-mercapto-substituted-4(3*H*)-quinazolinones (**A**) are reported for *h*CA-I (KIs = 135–282 nM), *h*CA-II (KIs = 0.25–10.8 nM), *h*CA-IX (KIs of 3.7–50.4 nM), and CA-XII (KIs of 0.60–52.9 nM) as potent inhibitors (Alafeefy et al., 2016). Several other 2-[(3-substituted-4(3*H*)-quinazolinon-2-yl)thio]-*N*-(4-sulfamoylphenyl)acetamides (**B**) derivatives also showed potent inhibition of α-CA, from *Vibrio cholerae* (VchCA) and human “*h*CA-I” and “*h*CA-II,” at a nanomolar level (Alafeefy et al., 2014) (Figure 1). Recently, 2-[(3-Benzyl-6,7-dimethoxy-4-(3*H*)-quinazolinon-2-yl)thio]-*N*-(4-sulfamoylphenyl)propanamide was also reported for *h*CA-II (KI, 3.30 nM) inhibition (El-Azab et al., 2019).

In this study, we report the synthesis and *in-vitro* bovine carbonic anhydrase-II (*b*CA-II) and human carbonic anhydrase-II (*h*CA-II) inhibitory activities of a series of quinazolinone analogs. Furthermore, the mode of inhibition was further explored by kinetic studies of the active analogs. Additionally, molecular docking studies were carried out to predict the main structural features responsible for the anti-CA-II activities of the compounds to predict the structure-activity relationships.

## Experimental

### Chemistry

The chemicals used in this study were purchased from Sigma and Aldrich in extra purified form. Melting points for all compounds were recorded by Büchi 434 melting point apparatus. FT-IR spectra (KBr disks) were measured on a Bruker FT-IR IFS48 spectrophotometer. NMR spectra were recorded on Bruker Avance 400 spectrometers in DMSO-d^6^. The chromatograms were visualized under UV light irradiation. The CHN analysis was performed on a Carlo Erba Strumentazione-Mod-1106.

#### General Procedure for Synthesis of 2-substituted-4*H*-benzo[*d*][1,3]oxazin-4-ones (3a-p) (Bari et al., 2020)

To an 100 mL round bottom flask, anthranilic acid **1** (0.01 mol) and dry pyridine (30 mL) were added at room temperature with stirring. The solution was cooled to 0°C, followed by dropwise addition of the corresponding aromatic acid chloride **2a-p** (0.02 mol) in 10 mL of dry pyridine with constant stirring. After this addition, the reaction mixture was stirred for a further half an hour at room temperature and set aside for 1 h. The product, obtained as a pasty mass, was diluted with ice cold water (50 mL) and treated with aqueous sodium bicarbonate solution. When the effervescence ceased, the precipitate obtained was filtered and washed with water. The crude benzoxazine obtained was dried and recrystallized from aqueous ethanol.

#### General Procedure for the Synthesis of Quinazolinone (4a-p) (Panicker et al., 2010)

To a 50 mL round bottom flask fitted with reflux condenser were added 2-substituted-4*H*-benzo[*d*][1,3]oxazin-4-one (0.01 mol) and hydrazine hydrate (0.06 mol) in excess. The reaction mixture was heated under reflux in an oil bath for 10–16 h. The course of the reaction was monitored by thin layer chromatography (TLC). After completion of the reaction as checked by TLC, the reaction mixture was cooled and separated solids were collected by filtration, washed with water/hexane, dried, and purified by column chromatography (methanol/chloroform/water, 10:2:1) to afford the corresponding quinazolinones **4a-p**.

### Experimental Data

#### 3-Amino-2-Phenylquinazolin-4(3*H*)-one (4a) (Panicker et al., 2010)

Yield: 82%; mp: 200–202°C; FT-IR (υ_max_, KBR, cm^−1^): 3,319, 3,051, 1,660, 1,603, 1,490, 1258; ^1^H NMR (400 MHz, DMSO-d^6^, δ, ppm): 5.36 (s, 2H), 7.47–8.18 (m, 9H); Anal. Calcd. for C_14_H_11_N_3_O: C, 70.88; H, 4.64; N, 17.72. Found: C, 70.92; H, 4.67; N, 17.68.

#### 3-Amino-2-(Napthalen-1-yl) Quinazolin-4(3*H*)-one (4b)

Yield: 80%; m.p: 215–216°C; FT-IR (υ_max_, KBR, cm^−1^): 3,316, 3,053, 1,662, 1,600, 1,493, 1,257; ^1^H NMR (400 MHz, DMSO-d^6^, δ, ppm): 5.42 (2H, s) 7.55–7.64 (4H, m), 7.7–7.88 (4H, m), 8.20 (1H, d, *J* = 7.4 Hz),), 8.42 (1H, d, *J* = 7.2 Hz), 8.38 (1H, d, *J* = 7.2 Hz); Anal. Calcd. for C_14_H_11_N_3_O: C, 70.88; H, 4.64; N, 17.72. Found: C, 70.92; H, 4.67; N, 17.68.

#### 3-Amino-2-(2-Nitrophenyl) Quinazolin-4(3*H*)-one (4c) (Pandit and Dodiya, 2013)

Yield: 77%; m.p: 180–182°C; FT-IR (υ_max_, KBR, cm^−1^): 3,309, 3,053, 1,673, 1,603, 1,548, 1,489, 1,354, 1,250; 701; ^1^H NMR (400 MHz, DMSO-d^6^, δ, ppm): 5.43 (2H, s), 7.51–7.63 (4H, m), 7.91–8.02 (4H, m); Anal. Calcd. for C_14_H_10_N_4_O_3_: C, 59.57; H, 3.57; N, 19.85; Found: C, 59.56; H, 3.58; N, 19.85.

#### 3-Amino-2-(3-Nitrophenyl) Quinazolin-4(3*H*)-one (4d) (Pandit and Dodiya, 2013)

Yield: 84%; m.p: 206–208°C; FT-IR (υ_max_, KBR, cm^−1^): 3,308, 3,053, 1,675, 1,600; 1,548, 1,490, 1,248, 1,355, 703; ^1^H NMR (400 MHz, DMSO-d^6^, δ, ppm): 5.48 (s, 2H), 7.62–7.68 (3H, m), 8.07 (1H, d, *J* = 7.4 Hz), 8.2 (1H, d, *J* = 7.2 Hz), 8.27 (1H, d, *J* = 7.8 Hz), 8.56 (1H, s); Anal. Calcd. for C_14_H_10_N_4_O_3_: C, 59.57; H, 3.57; N, 19.85. Found: C, 59.56; H, 3.58; N, 19.85.

#### 3-Amino-2-(4-nitrophenyl) quinazolin-4(3*H*)-one (4e) (Pandit and Dodiya, 2013)

Yield: 76%; m.p: 224–225°C; FT-IR (υ_max_, KBR, cm^−1^): 3,307, 3,053, 1,670, 1,603, 1,548, 1,488, 1,353, 1,250; 708; ^1^H NMR (400 MHz, DMSO-d^6^, δ, ppm): 5.42 (2H, s), 7.66–7.71 (3H, m), 8.06 (1H, d, *J*= 7.2 Hz), 8.12 (2H, d, *J* = 7.8 Hz), 8.26 (2H, d, *J* = 7.8 Hz); Anal. Calcd. for C_14_H_10_N_4_O_3_: C, 59.57; H, 3.57; N, 19.85. Found: C, 59.56; H, 3.58; N, 19.85.

#### 3-Amino-2-(2-Bromophenyl) Quinazolin-4(3*H*)-one (4f)

Yield: 79%; m.p: 204–206°C; FT-IR (υ_max_, KBR, cm^−1^): 3,300, 3,053, 1,673, 1,610, 1,010, 690; ^1^H NMR (400 MHz, DMSO-d^6^, δ, ppm): 5.25 (2H, s), 7.35–7.39 (2H, t, *J* = 7.2 Hz), 8.06 (1H, d, *J* = 8.1 Hz), 7.58–7.66 (5H, m); *Anal*. Calcd for C_14_H_10_BrN_3_O: C, 53.19; H, 3.19; N, 13.29. Found: C, 53.17; H, 3.20; N, 13.28.

#### 3-Amino-2-(4-Bromophenyl) Quinazolin-4(3*H*)-one (4g)

Yield: 81%; m.p: 184–185°C; FT-IR (υ_max_, KBR, cm^−1^): 3,300, 3,053, 1,676, 1,610, 1,010, 690; ^1^H NMR (400 MHz, DMSO-d^6^, δ, ppm): 5.26 (2H, s), 7.52 (2H, d, *J* = 7.45 Hz), 7.61–7.67 (3H, m), 7.74 (2H, d, *J* = 7.6 Hz), 8.1 (1H, d, *J* = 7.55 Hz); *Anal*. Calcd for C_14_H_10_BrN_3_O: C, 53.19; H, 3.19; N, 13.29. Found: C, 53.17; H, 3.20; N, 13.28.

#### 3-Amino-2-(4-Methylphenyl) Quinazolin-4(3*H*)-one (4h) (Babu et al., 2014)

Yield: 85%; mp: 294–296°C; FT-IR (υ_max_, KBR, cm^−1^): 3,300, 3,053, 2,948, 1,676, 1,610, 1,496; ^1^H NMR (400 MHz, DMSO-d^6^, δ, ppm): 2.34 (3H, s), 4.93 (2H, s), 8.08 (1H, d, *J* = 7.2 Hz), 7.28 (2H,d, *J* = 7.8 Hz), 7.44 (2H, d, *J* = 7.8 Hz), 7.55-7.67 (3H, m); *Anal*. Calcd for C_15_H_13_N_3_O: C, 71.70; H, 5.21; N, 16.72. Found: C, 71.71; H, 5.20; N, 16.73.

#### 3-Amino-2-(2-Fluorophenyl) Quinazolin-4(3*H*)-one (4i)

Yield: 84%; m.p: 169–170°C; (υ_max_, KBR, cm^−1^): 3,308, 3,053, 1,681, 1,610; 1,329, 506 ^1^H NMR (400 MHz, DMSO-d^6^, δ, ppm): 4.98 (2H, s), 8.20 (1H, d, *J* = 7.2 Hz), 7.77 (1H, bd, *J* = 8.4 Hz), 7.59–7.67 (4H, m), 7.29–7.36 (2H, m); *Anal*. Calcd for C_14_H_10_FN_3_O: C, 65.88; H, 3.95; N, 16.46. Found: C, 65.89; H, 3.94; N, 16.47

#### 3-Amino-2-(3-Fluorophenyl) Quinazolin-4(3*H*)-one (4j)

Yield: 78%; m.p: 164–165°C; FT-IR (υ_max_, KBR, cm^−1^): 3,310, 3,053, 1,679, 1,610; 1,335, 502; ^1^H NMR (400 MHz, DMSO-d6, δ, ppm): 5.52 (2H, s), 8.01 (1H, d, *J* = 7.2Hz), 7.78 (1H, d, *J* = 7.8 Hz), 7.58–7.66 (4H, m), 7.32 (1H, t, *J* = 7.8 Hz); *Anal*. Calcd for C_14_H_10_FN_3_O: C, 65.88; H, 3.95; N, 16.46. Found: C, 65.89; H, 3.94; N, 16.47.

#### 3-Amino-2-(4-Fluorophenyl) Quinazolin-4(3*H*)-one (4k)

Yield: 81%; m.p: 176–178°C; FT-IR (υ_max_, KBR, cm^−1^): 3,310, 3,053, 1,680, 1,610; 1,335, 502; ^1^H NMR (400 MHz, DMSO-d^6^, δ, ppm): 5.38 (s, 2H), 7.26–7.30 (2H, m), 7.41–7.46 (2H, m), 7.53–7.56 (2H, m), 8.26 (1H,d, *J* = 7.5 Hz); *Anal*. Calcd for C_14_H_10_FN_3_O: C, 65.88; H, 3.95; N, 16.46. Found: C, 65.87; H, 3.94; N, 16.47.

#### 3-Amino-2-(2-Chlorophenyl) Quinazolin-4(3*H*)-one (4l) (Pandit and Dodiya, 2013)

Yield: 85%; m.p: 160–162°C; FT-IR (υ_max_, KBR, cm^−1^): 3,310, 3,053, 1,689, 1,610; ^1^H NMR (400 MHz, DMSO-d^6^, δ, ppm): 5.08 (2H, s), 7.41–7.45 (2H, t, *J* = 7.3 Hz), 7.48 (1H, d, *J* = 7.3 Hz) 7.54 (1H, d, *J* = 7.3 Hz), 7.58–7.69 (3H, m), 8.07 (1H, d, *J* = 7.2 Hz); *Anal*. Calcd for C_14_H_10_ClN_3_O: C, 61.89; H, 3.71; N, 15.47. Found C, 61.87; H, 3.73; N, 15.47.

#### 3-Amino-2-(3-Chlorophenyl) Quinazolin-4(3*H*)-one (4m) (Pandit and Dodiya, 2013)

Yield: 79%; m.p: 144–145°C; FT-IR (υ_max_, KBR, cm^−1^): 3,307, 3,210, 3,051, 2,993, 1,668, 1,591, 1,252, 1,123; 458; ^1^H NMR (400 MHz, DMSO-d^6^, δ, ppm): 5.33 (2H, s), 8.14 (1H, d, *J* = 7.2 Hz), 7.84 (1H, s), 7.63–7.68 (3H, m), 7.46–7.52 (3H, m); *Anal*. Calcd for C_14_H_10_ClN_3_O: C, 61.89; H, 3.71; N, 15.47. Found C, 61.87; H, 3.73; N, 15.47.

#### 3-Amino-2-(4-Chlorophenyl) Quinazolin-4(3*H*)-one (4n) (Castillo et al., 2012)

Yield: 86%; m.p: 165–166°C; FT-IR (υ_max_, KBR, cm^−1^): 3,310, 3,213, 3,053, 2,990, 1,670, 1,591, 1,252, 1,123; 462 ^1^H NMR (400 MHz, DMSO-d^6^, δ, ppm): 4.86 (s, 2H) 7.34 (2H, d, *J* = 7.4 Hz), 7.56 (2H, d, *J* = 7.4 Hz), 7.68–7.75 (3H, m), 8.04 (1H, d, *J* = 7.2 Hz); Anal. Calcd for C_14_H_10_ClN_3_O: C, 61.89; H, 3.71; N, 15.47. Found C, 61.87; H, 3.73; N, 15.47.

#### 3-Amino-2-(4-Methoxyphenyl) Quinazolin-4(3*H*)-one (4o) (Pandit and Dodiya, 2013)

Yield: 88%; m.p: 221–223°C; FT-IR (υ_max_, KBR, cm^−1^): 3,310, 3,053, 2,940, 1,689, 1,610, 1,490; ^1^H NMR (400 MHz, DMSO-d^6^, δ, ppm): 4.92 (2H,s), 3.76 (3H, s), 6.96 (2H, d, *J* =7.5 Hz), 7.34 (2H, d, *J* = 7.5 Hz),7.60–7.68 (3H, m), 8.09 (1H, d, *J* = 7.3 Hz); Anal. Calcd. for C_15_H_13_N_3_O_2_: C, 67.40; H, 4.90; N, 15.72; Found C, 67.42; H, 4.88; N, 15.71.

#### 3-Amino-2-(3,4,5-Trimethoxyphenyl) Quinazolin-4(3*H*)-one (4p)

Yield: 78%; m.p: 221–222°C; FT-IR (υ_max_, KBR, cm^−1^): 3,310, 3,053, 2,950, 1,689, 1,610, 1,495; ^1^H NMR (400 MHz, DMSO-d^6^, δ, ppm): 3.79 (6H, s), 3.82 (3H, s), 4.88 (2H, s), 6.62 (2H, s), 7.48–7.52 (3H, m), 8.01 (1H, d, *J* = 7.3 Hz); Anal. Calcd. for C_17_H_17_N_3_O_4_: C, 62.38; H, 5.23; N, 12.84; Found C, 62.38; H, 5.24; N, 12.83.

### *In-vitro* Assay Protocol

*In-vitro b*CA-II and *h*CA-II activities were measured by following the spectrophotometric method described by Pocker and Meany with slight modifications (Pocker and Meany, 1967; Ur Rehman et al., 2020). The spectrophotometric assay was conducted in HEPES-Tris buffer of pH 7.4 (20 mM) at 25°C. Each inhibitory well-consisted of 140 μL of HEPES-Tris buffer solution, 20 μL of *b*CA-II enzyme solution (0.1 mg/mL HEPES-Tris buffer), and 20 μL of test compound in HPLC grade DMSO (maintain 10% of the final concentration). The mixture solution was pre-incubated for 15 min at 25°C. Substrate *p*-nitrophenyl acetate (*p*-NPA) (0.7 mM) was prepared in HPLC grade methanol and the reaction was started by adding 20 μL to a well in a 96-well-plate. The amount of product formed was measured for 30 min continuously at 1 min intervals at 400 nm in a 96-well-plate using xMARK microplate spectrophotometer, Bio-Rad (USA). The activity of the controlled compound was taken as 100%. All experiments were carried out in triplicates of each used concentration, and results are represented as a mean of the triplicate.

### Molecular Docking Protocol

The docking studies were performed on the crystal structures of *b*CA-II (1V9E, resolution = 1.95 Å) (Saito et al., 2004) and *h*CA-II (1BN1, resolution = 2.1 Å) (Boriack-Sjodin et al., 1998). The selected target receptors were retrieved from Protein Data Bank (www.rcsb.org) in PDB format. Chain A of each protein (1V9E and 1BN1) was chosen for docking. For the molecular docking simulation, Molecular Operating Environment (MOE) docking suite was used (MOEv2014.09). The crystal structures were prepared using Protein Preparation Wizard implemented in the MOE with the default settings. Hydrogen atoms were added in the MOE using protonate 3D protocol and the protein structures were minimized with an MMFF94x force field until an RMSD gradient of 0.1 kcal·mol^−1^Å^−1^ was achieved. The 3D-structures of ligands were prepared by Chemdraw and minimized by default option of MOE (Force field = MMFF94x, RMS gradient = 0.1 kcal·mol^−1^Å^−1^). For docking, Triangle Matcher placement method and London dG scoring function were applied (Edelsbrunner, 1992; Naïm et al., 2007). After docking, thirty docked poses of each compound were saved and the best-scoring docked pose of each ligand was visualized.

## Results and Discussion

### Chemistry

**Scheme 1:**
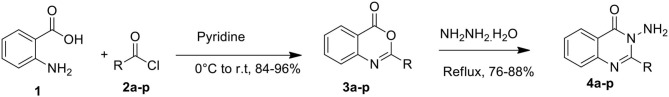
Synthesis of quinazolinone derivatives.

The target compounds were synthesized according to the route depicted in Scheme 1. Anthranilic acid **1** was treated with corresponding acid chlorides (**2a-p**) in the presence of pyridine to form precursor heterocycle (**3a-p**) via literature method (Scheme 1) (Bari et al., 2020). The corresponding benzoxazinones were refluxed in excess of hydrazine hydrate to furnish the 3-Amino-2-aryl quinazolin-4(3*H*)-ones (**4a-p**) in moderate to excellent yields of 76–88% (Table 1) (Panicker et al., 2010).

**Table 1:** Different analogs, % yield, and reaction time of quinazolinones.

**Entry**	**Compounds**	**R**	**% Yields**	**Reaction time(h)**
1	**4a**	Phenyl	82	12
2	**4b**	1′- Naphthyl	80	10
3	**4c**	2′-Nitrophenyl	77	16
4	**4d**	3′-Nitrophenyl	84	20
5	**4e**	4′-Nitrophenyl	76	20
6	**4f**	2′-Bromophenyl	79	11
7	**4g**	4′-Bromophenyl	81	12
8	**4h**	4′-Methylphenyl	85	8
9	**4i**	2′-Fluorophenyl	84	12
10	**4j**	3′-Fluorphenyl	78	14
11	**4k**	4′-Fluorophenyl	81	10
12	**4l**	2′-Chlorophenyl	85	10
13	**4m**	3′-Chlorophenyl	79	12
14	**4n**	4′-Chlorophenyl	86	10
15	**4o**	4′-Methoxyphenyl	88	15
16	**4p**	3′,4′,5′-Trimethoxyphenyl	78	13

The structures of compounds (**4a-p**) were established using microanalysis (CHN) and spectral data, i.e., IR and ^1^H NMR. The C=N band in FTIR appeared in the range of 1,489–1,521 cm^−1^. Moreover, the carbonyl group of compounds (**4a-p**) appeared at 1,660–1,689 cm^−1^. The amino moiety (-NH_2_) of compounds (**4a-p**) was verified by the characteristic peak at 3,300–3,319 cm^−1^ in FT-IR spectra. The amino-moiety of compounds (**4a-p**) was verified by ^1^H NMR spectra which appeared as a singlet for amino protons in a range from δ 4.92–5.52 *ppm*. The spectral data of other aromatic and aliphatic protons was also in accordance with the structures of anticipated compounds. CHN analysis also supported the anticipated structures (**4a-p**) and the observed values were in good agreement with the values found.

### Biology

#### *In-vitro* bCA-II and *h*CA-II inhibitions

All quinazolinone analogs **(4a-p)** were evaluated against bovine carbonic anhydrase-II (*b*CA-II) and human carbonic anhydrase-II (*h*CA-II) for their ability to act as an inhibitor of CA-II. All the assays were carried out at a micromolar level using acetazolamide as a standard inhibitor. After initial screening, all the analogs demonstrated significant inhibitory activity against *b*CA-II with IC_50_ values in the range of 8.9 ± 0.31 −67.3 ± 0.42 μM, except compounds **4i** and **4o**, which were found to be inactive (Table 2). Moreover, compounds **4c**, **4e**, **4f**, **4l**, and **4m** showed more superior activity than the standard drug ‘acetazolamide' (IC_50_ = 18.2 ± 0.43 μM). Compound **4m** was the most active inhibitor (IC_50_ = 8.9 ± 0.31 μM), followed by **4e (**IC_50_ = 9.1 ± 0.21 μM), **4l (**IC_50_ = 10.7 ± 0.82 μM), **4c (**IC_50_ = 11.7 ± 0.36 μM), and **4f (**IC_50_ = 17.9 ± 0.56 μM). However, compounds **4d**, **4k**, **4n**, and **4p** depicted biological activity comparable to the standard acetazolamide with IC_50_ values in range of 18.3 ± 0.51–28.2 ± 0.01 μM, while compounds **4a**, **4b**, **4g**, **4h**, and **4j** were found to be the least active hits of the series. For *h*CA-II, compounds **4a**, **4c**-**4h**, and **4o** were found to be active with IC_50_ values in the range of 14.0 ± 0.60–59.6 ± 1.03 μM, as compared to acetazolamide (IC_50_ = 19.6 ± 1.23 μM) (Table 2). Among all the compounds, **4g** (IC_50_ = 14.0 ± 0.60 μM) exhibited better activity than the standard drug, while compounds **4c** (IC_50_ = 21.1 ± 1.36 μM), **4d** (IC_50_ = 21.5 ± 0.52 μM), and **4o** (IC_50_ = 22.0 ± 0.40 μM) demonstrated activities comparable to acetazolamide. Compounds **4e** (IC_50_ = 39.9 ± 2.21 μM), **4f** (IC_50_ = 43.5 ± 1.51 μM), **4h** (IC_50_ = 53.0 ± 2.12 μM), and **4a** (IC_50_ = 59.6 ± 1.03 μM) were shown to be the least active hits in this series. The *in-vitro* results indicated that compounds **4g** and **4o** are more selective inhibitors for *h*CA-II. The biological activities of all the compounds are tabulated in Table 2.

**Table 2:** *In-vitro* inhibition results of compounds (**4a-p**) against *b*CA-II and *h*CA-II.

**Bovine carbonic anhydrase-II (*****b*****CA-II)**			**Human carbonic anhydrase-II (*****h*****CA-II)**	
**Compounds**	**% Inhibition (0.5 mM)**	**IC_**50**_ ± SEM (μM)**	**% Inhibition (0.5 mM)**	**IC_**50**_ ± SEM (μM)**
**4a**	75.2	53.6 ± 0.85	75.2	59.6 ± 1.03
**4b**	83.6	67.3 ± 0.42	38.1	NA
**4c**	80.5	11.7 ± 0.36	88.5	21.1 ± 1.36
**4d**	92.9	19.7 ± 1.02	94.2	21.5 ± 0.52
**4e**	80.7	9.1 ± 0.21	92.7	39.9 ± 2.21
**4f**	88.9	17.9 ± 0.56	98.9	43.5 ± 1.51
**4g**	85.3	33.6 ± 0.22	85.3	14.0 ± 0.60
**4h**	92.4	38.0 ± 0.62	92.4	53.0 ± 2.12
**4i**	17.6	NA	27.8	NA
**4j**	78.6	46.6 ± 0.39	28.1	NA
**4k**	76.3	18.3 ± 0.51	36.2	NA
**4l**	78.2	10.7 ± 0.82	28.7	NA
**4m**	60.0	8.9 ± 0.31	30.5	NA
**4n**	54.2	28.2 ± 0.01	24.3	NA
**4o**	19.9	NA	89.4	22.0 ± 0.40
**4p**	87.3	26.8 ± 0.23	27.4	NA
**Acetazolamide**	86.4	18.2 ± 0.43	83.2	19.6 ± 1.23

SEM, Standard error mean; NA, Not active.

#### Kinetics Studies

**Figure 2:**
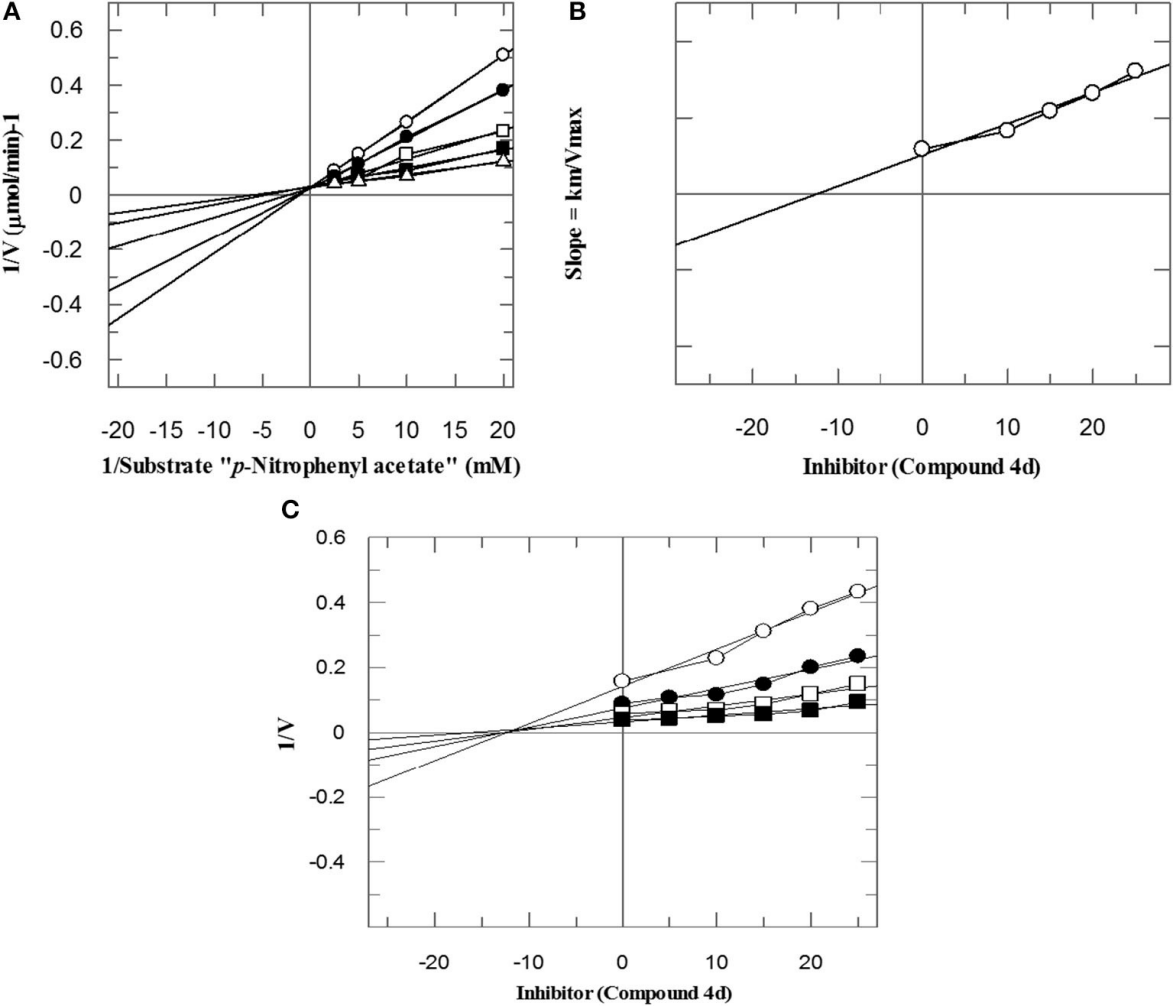
The mode of inhibition of bCA-II by compound **4d**. **(A)** Lineweaver–Burk plot of reciprocal rate of reaction (velocities) vs. reciprocal of substrate (*p*-nitrophenyl acetate) in the absence (Δ), and in the presence of 14 μM (■), 17 μM (□), 20 μM (•), and 23 μM (∘) of compound **4d**. **(B)** Secondary re-plot of Lineweaver–Burk plot between the slopes of each line on Lineweaver–Burk plot vs. different concentrations of compound **4d**. **(C)** Dixon plot of reciprocal rate of reaction (velocities) vs. different concentrations of compound **4d**.

**Figure 3:**
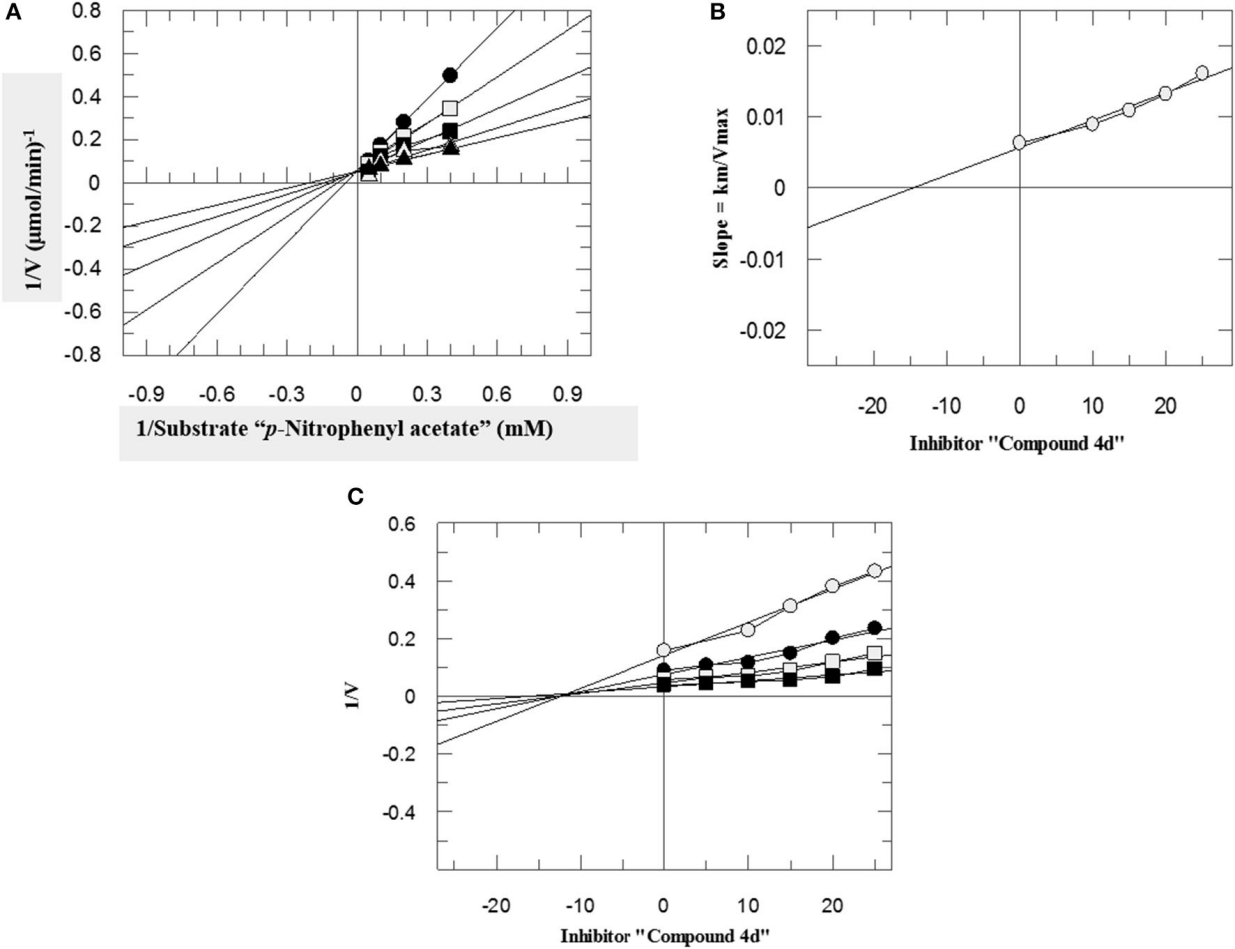
The mode of inhibition of *h*CA-II by compound **4d**. **(A)** Lineweaver–Burk plot of reciprocal rate of reaction (velocities) vs. reciprocal of substrate (p-nitrophenyl acetate) in the absence (▴), and in the presence of 10 μM (Δ), 15 μM (■), 20 μM (□), and 25 μM (•) of compound **4d**. **(B)** Secondary re-plot of Lineweaver–Burk plot between the slopes of each line on Lineweaver–Burk plot vs. different concentrations of compound **4d**. **(C)** Dixon plot of reciprocal rate of reaction (velocities) vs. different concentrations of compound **4d**.

To investigate the mechanism of action of these compounds, kinetics studies were performed. The kinetics studies were used to discover the type of inhibition and dissociation constant (*Ki*). Kinetics studies of the most active compound, **4d**, against both *b*CA-II and *h*CA-II were performed, using different substrate concentrations on one side with different concentrations of **4d** on the other side. Compound **4d** inhibited both the *b*CA-II and *h*CA-II enzymes in a concentration-dependent manner with *Ki* values of 13.0 ± 0.013 and 14.25 ± 0.017 μM, respectively. From the kinetics studies, it was deduced that the compound **4d** is a competitive inhibitor for both *b*CA-II and *h*CA-II. The Lineweaver-Burk plots were used for determination of the type of inhibition, in which the reciprocal of substrate concentrations was plotted against the reciprocal of the rate of the reaction to monitor the effect of the inhibitor on both *K*_*m*_ and *V*_*max*_. The Lineweaver-Burk plots of **4d** against both *b*CA-II and *h*CA-II clearly showed that the mode of inhibition of **4d** is competitive (Figures 2A, 3A). In competitive inhibition, the *K*_*m*_ of enzyme increased, while *V*_*max*_ are not affected. The Lineweaver-Burk plots of compounds **4d** (Figures 2A, 3A) showed that in the presence of compounds **4d**, the *K*_*m*_ of both enzymes *b*CA-II and *h*CA-II increased significantly on applying compounds **4d**, while the *V*_*max*_ remain unchanged, which described the competitive behavior of compounds **4d** and its interaction in the active site of the enzyme.

The *K*_*i*_ values of compounds **4d** for both enzymes *b*CA-II and *h*CA-II were deduced by secondary replots of Lineweaver-Burk plots by plotting the slope of each line in the Lineweaver-Burk plots against different concentrations of compound **4d** (Figures 2B, 3B). The *K*_*i*_ values were confirmed by Dixon plot after plotting the reciprocal of the rate of reaction against different concentrations of compound **4d** (Figures 2C, 3C).

### Computational Studies

Computational medicinal chemistry has expedited the pace of drug design and discovery over the last four decades (Lin et al., 2020). Docking is a computational method which is widely used to study and understand the interaction between two macro-molecules (for e.g., protein-protein or protein-DNA) or between a macromolecule and ligand (e.g., for a drug-receptor, drug-DNA, or drug-RNA) and effectively predict the inhibitory mechanism of drugs. Therefore, we predicted the mode of interactions of all the active compounds though molecular docking. The reference inhibitor (acetazolamide) and quinazolinones were docked into the catalytic active pocket of *h*CA-II and *b*CA-II. The binding modes and receptor-ligand interactions in the binding site of *b*CA-II and *h*CA-II in the three-dimensional and two-dimensional form were carefully examined by visual analysis through MOE. The results are presented in Tables 3, 4. Molecular docking revealed that all the active quinazolinones derivatives were exactly fitted into the active catalytic pocket of both enzymes (*h*CA-II and *b*CA-II). The docked orientation of compounds revealed direct interactions of ligands with the zinc ion present in the active site. Moreover, interaction of compounds with the active site residues and water molecule stabilize the compounds in the active site.

**Table 3:** Docking results of all active compounds against *b*CA-II.

**Compounds**	**Docking** **score**	**Ligand** **atoms**	**Receptor** **atoms**	**Interaction** **type**	**Distance** **(Å)**
**4m**	−5.50	O19 N18 N9	ZN OG1-THR198 NE2-GLN91	Metallic HBD HBA	3.48 3.19 1.38
**4e**	−5.29	O19 N7 N18 N9	ZN HOH310 OG1-THR198 NE1-GLN91	Metallic HBA HBA HBA	3.58 2.80 3.09 3.17
**4l**	−5.69	O19 N7 N9	ZN HG1-THR198 NE2-GLN91	Metallic HBA HBA	3.47 1.98 3.06
**4c**	−6.14	O19 N20 N7 O22	ZN ND2-ASN66 OG1-THR198 NE1-GLN92	Metallic HBA HBA HBA	3.16 2.77 2.96 3.02
**4f**	−5.70	O19 N7 N18	ZN HOH493 HOH279	Metallic HBA HBA	3.51 2.93 1.27
**4k**	−5.36	O19 N7 N9	ZN OG1-THR198 OE2-GLN91	Metallic HBA HBA	3.44 1.96 3.07
**4d**	−5.60	O19 N7	ZN OG1-THR199	Metallic HBA	3.48 1.38
**4p**	−5.86	O19 N9 N18	ZN NE2-GLN91 OG1-THR198	Metallic HBD HBA	3.43 3.38 2.55
**4n**	−5.27	O19 N18 N9	ZN OG1-THR198 NE2-GLN91	Metallic HBD HBA	3.54 1.77 3.17
**4g**	−5.17	O11 6-ring	OG1-THR198 ND2-ASN66	HBA Pi-H	2.81 3.50
**4h**	−5.25	N19	OG1-THR198	HBA	2.99
**4j**	−5.72	N19 O11	HOH-340 N-THR197	HBD HBA	2.92 2.85
**4a**	−5.38	O11	OG1-THR198	HBA	2.07
**4b**	−4.99	N22 O11	NE1-GLN91 HOH-435	HBD HBA	3.11 2.65
**Standard (Acetazolamide)**	−5.17	N5 O10 N6	ZN NE1-GLN91 HOH463	Metallic HBA HBD	3.35 3.47 1.33

*HBA, Hydrogen bond acceptor; HBD, Hydrogen bond donor*.

**Table 4:** Computational analysis of all active compounds against *h*CA-II.

**Code**	**Docking score**	**Ligand atoms**	**receptor Atoms**	**interaction type**	**Distance (Å)**
**4g**	−4.51	N18 N18 N18	HOH270 ND2-ASN62 OG1-THR200	HBD HBA HBA	2.45 3.34 2.99
**4c**	−4.96	O20 N18	NE2-GLN92 HOH270	HBA HBA	3.42 2.78
**4d**	−5.36	O21 N11	ZN OG1-THR200	Metallic HBA	2.73 2.68
**4o**	−5.31	O20 N7 N18	ZN NE2-GLN92 OG1-THR200	Metallic HBA HBD	2.71 2.79 3.38
**4e**	−5.20	O20 N7	ZN NE2-GLN92	Metallic HBA	2.54 2.65
**4f**	−4.43	N18 O19	OG1-THR200 HOH270	HBD HBA	3.10 2.89
**4h**	−4.73	N18 O19	OG1-THR200 HOH270	HBA HBA	3.33 2.38
**4a**	−4.44	O19 N18	ND2-ASN62 OD1-ASN67	HBD HBA	1.89 2.74
**Standard (Acetazolamide**)	−4.85	O10 O11 O11	ZN OG1-THR199 OG1-THR-200	Metallic HBA HBD	2.73 3.12 3.60

*HBA, Hydrogen bond acceptor; HBD, Hydrogen bond donor*.

**Figure 4:**
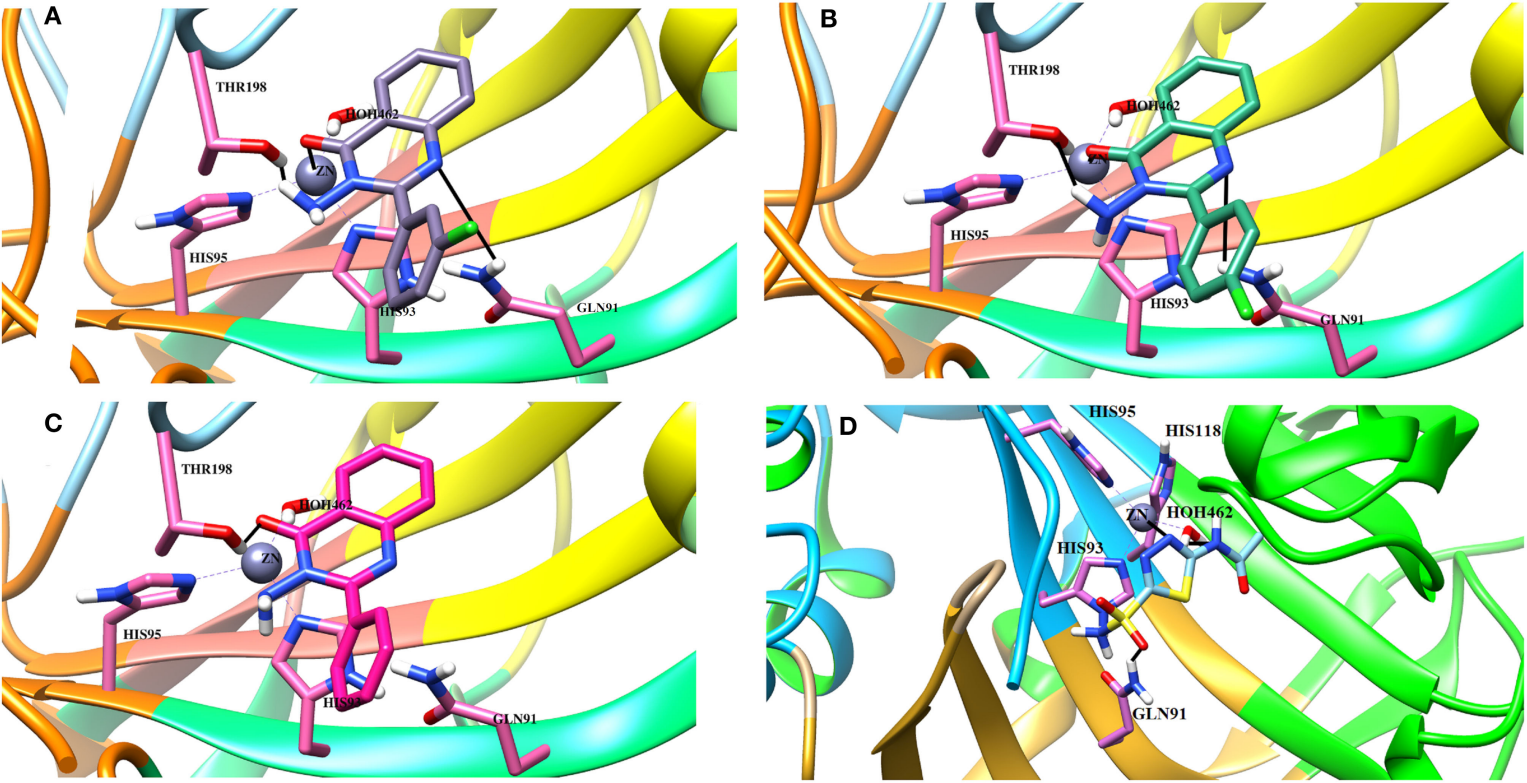
The 3D interaction of the **(A)** most active compound (**4 m**, shown in gray stick model), **(B)** moderate active compound (**4n**, green stick model), **(C)** least active compound (**4a**, pink stick model), and **(D)** standard drug (acetazolamide, shown in cyan sticks) in the active site of bCA-II. The active site residues are depicted in pink stick model. The hydrogen bonds are presented in black lines.

The docking process was first validated by re-docking the standard inhibitor acetazolamide in the active site of enzyme and is presented in Figures 4D, **6D**.

#### Binding Interactions of Active Compounds and Their Predictive SAR Against *b*CA-II

From the docked poses of the all active compounds, it was confirmed that the compounds directly interact with Zn^2+^ ion through their quinazolinone-carbonyl moiety. Compounds **4c**, **4d**, **4e**, **4f**, **4k**, **4l**, and **4m** were found to be potent inhibitors of *b*CA-II with IC_50_ values in the range of 8.9–19.7 μM. Compound **4m** was the most potent inhibitor (IC_50_ = 8.9 ± 0.31μM), followed by **4e** > **4l** > **4c** > **4f** > **4k** > **4d**, while compounds **4p** > **4n** displayed moderate active, and **4g** **>** **4h** **>** **4j** **>** **4a** > **4b** were retrieved as the least active, as compared to standard acetazolamide. The docked pose of **4m** indicates that the quinazolinone-carbonyl moiety of **4m** formed a metallic bond (3.44 Å) with the Zn^2+^ ion, while the quinazolinone-nitrogen formed hydrogen bonds with the side chain of Thr198 and Gln91 at a distance of 2.28 and 3.41 Å, respectively. Similarly, carbonyl oxygen and the nitro groups of **4e** mediated a metallic bond with Zn^2+^ ion (3.58 Å), and H-bonds with the side chains of Thr198 (2.80 Å) and Gln91 (3.09 Å). Additionally, the quinazolinone substituted amino group of **4e** mediated a H-bond with a water molecule in the active site (3.17 Å). The third most active compounds, **4l** and **4c**, also followed a similar type of interaction, however, **4c** was also stabilized by the side chains of Asn66 and Gln92 through hydrogen bonding. The carbonyl oxygen of **4f** and **4k** interacted with the Zn^2+^ ion, however **4f** lost the interactions with Thr198 and Gln91, instead mediating H-bonding with two water molecules (WAT493 and WAT279), however **4k** retained H-bonds with Thr198 and Gln91. Compound **4d** showed a metallic and a H-bond with the Zn^2+^ ion and Thr199, respectively, however it lost the interactions with Gln91 and water molecules.

The quinazolinone moiety of the moderate active compounds (**4p** and **4n**) interacted with the Zn^2+^ ion, while the quinazolinone moiety of both the compounds mediated hydrogen bonding with the side chains of Gln91 and Thr198.

**Figure 5:**
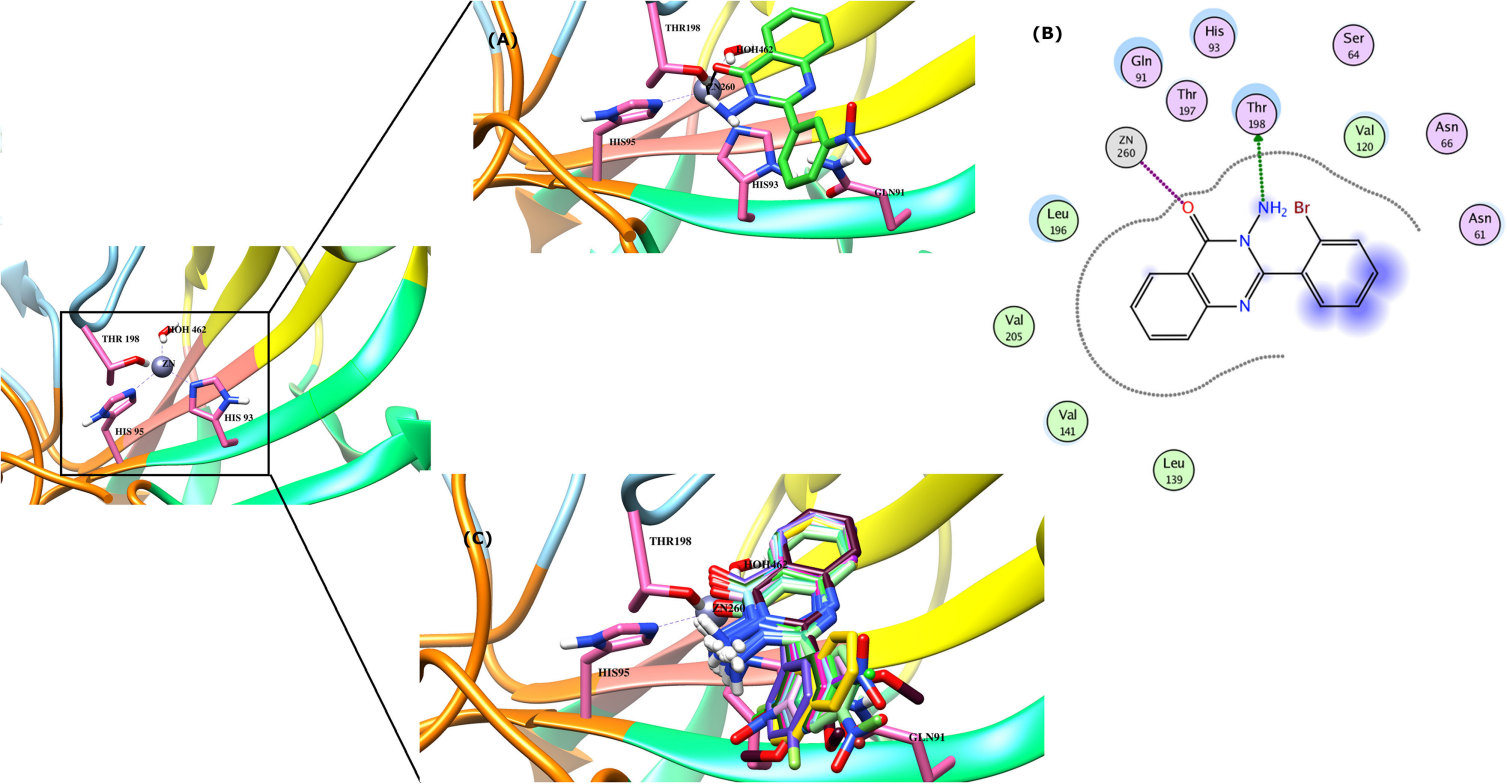
**(A)** The docked view of compound **4d** is shown in the active site of *b*CA-II in 3D-form. **(B)** The binding interactions of **4d** in two-dimensional form is demonstrated. **(C)** The binding modes of all the active compounds are shown. The hydrogen bonds are shown in black lines.

The docked view of the least active compounds (**4g**, **4h**, **4j**, **4a**, and **4b**) showed that quinazolinone carbonyl of **4g** interacted with the side chain of Thr198 via H-bond (2.81 Å) and the side chain of Asn66 offered a hydrophobic interaction to the compound (3.50 Å). Whereas, quinazolinone-amide group of **4h** mediated a H- bond with the side chain of Thr198 (2.99 Å), and the amide group of **4j** interacted with a water molecule (WAT340) present in the active site. Similarly, the carbonyl oxygen and amide nitrogen of compounds **4a** and **4b** mediated H-bonds with the side chain of Thr197 and Gln91, respectively. Additionally, **4b** formed a bond with a water molecule (WAT435). The binding interactions and docking scores of each docked compound are tabulated in Table 3. Figure 4 shows the binding interactions of the most active (**4m**), moderate (**4n**), and the least active (**4a**) compounds, however, a docked view of the most potent compound (**4d**) and all the compounds are presented in Figure 5.

#### Binding Interactions of Active Compounds and Their Predictive SAR Against *h*CA-II

**Figure 6:**
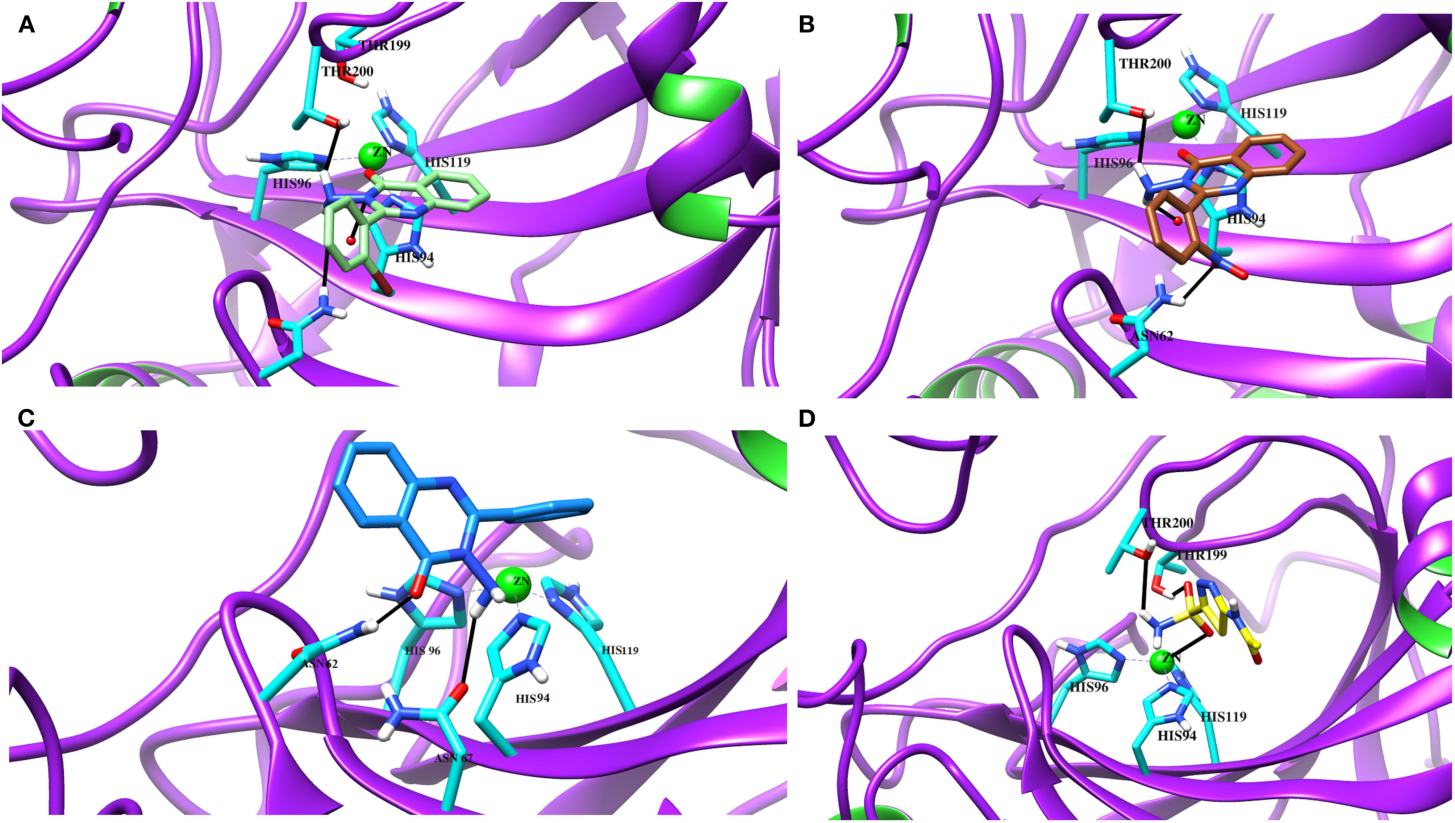
The 3D docked view of the **(A)** most active compound (**4g**, showed as lime green stick model), **(B)** moderate active compound (**4c**, shown as green stick model) **(C)** least active compound (**4a**, shown as sky blue stick model) and **(D)** standard (acetazolamide shown as yellow stick model) against hCA-II. The active site residues are depicted in cyan stick model. The hydrogen bonds are presented in black lines.

**Figure 7:**
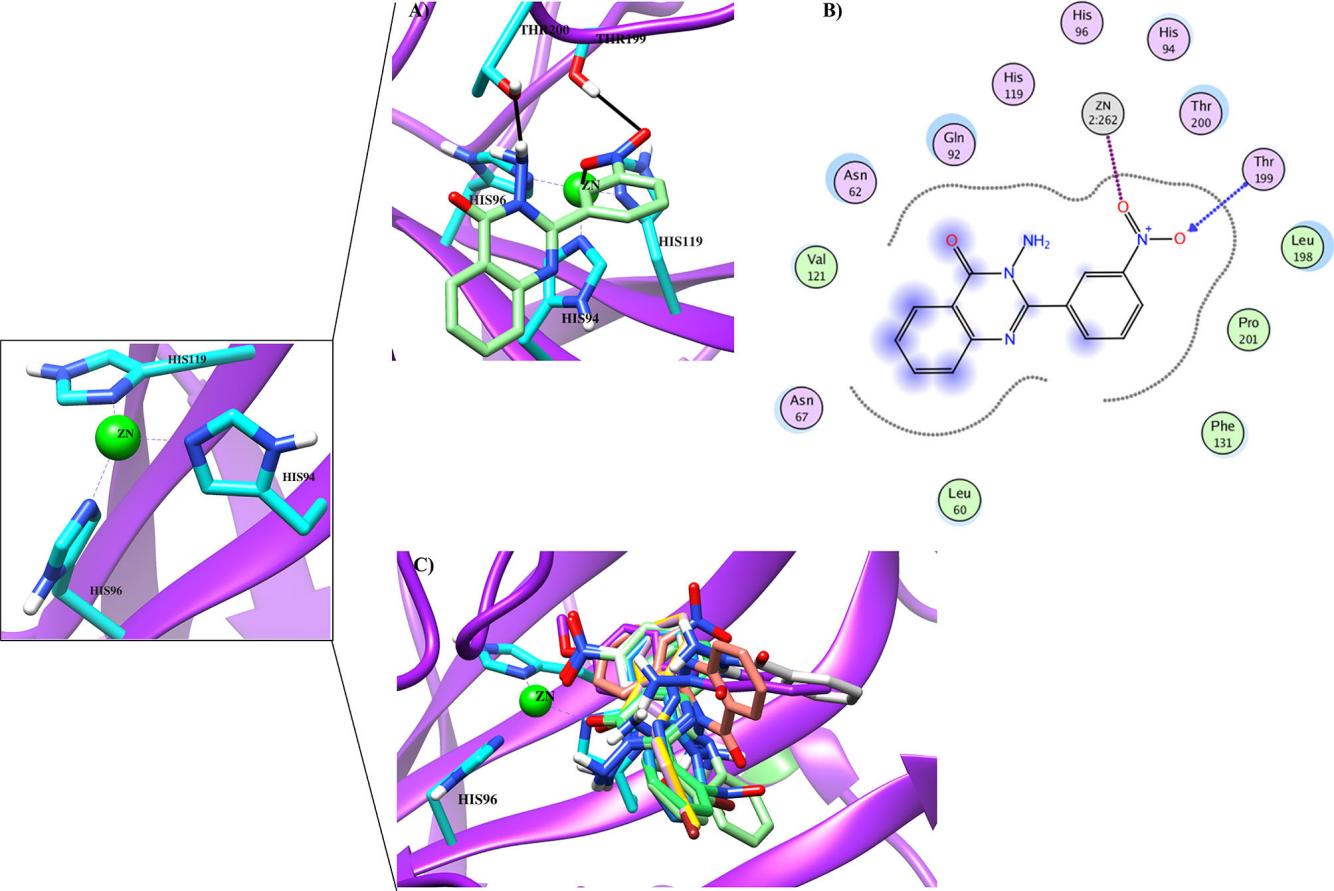
**(A)** The docked view of compound **4d** (lime green stick mode) in the active site of *h*CA-II. **(B)** The 2D-binding interactions of 4**d** are presented **(C)** The docked orientation of all the active compound in the active site of *h*CA-II are shown.

Docking analysis deduced that the quinazolinone moieties of these compounds are responsible for the formation of metallic interactions with the Zn^2+^ atom in the catalytic cavity. Compound **4g** (IC_50_ = 14.0 ± 0.60 μM) was the most potent and selective inhibitor of *h*CA-II, as compared to standard acetazolamide (IC_50_ = 19.6 ± 1.23 μM), followed by compounds **4c** > **4d** > **4o** and least active **4e** > **4f** > **4h** > **4a**. The quinazolinone moiety of **4g** interacted with the side chain of Asn62, Thr200, and Wat270 with bond lengths of 3.34, 2.99, and 2.45 Å, respectively. Similarly, **4c** interacted with the side chain of Gln92 and Wat270, whereas **4d** was linked with Zn^2+^ atom through its nitro-oxygen group. The nitro-oxygen and aminoquinoline moiety of **4o** interacted with Zn^2+^ atom and the side chains of Gln92 and Thr200 via hydrogen bonds. However, the quinazolinone moiety of **4e** interacted with the Zn^2+^ ion and Gln92. On the other hand, the least active compounds, **4a**, **4f**, and **4h**, interacted with the side chains of Asn62, Asn67, Thr200, and water molecules within the active site. The detailed binding interactions of active compounds and their docking scores are tabulated in Table 4. The binding interactions of most active (**4g**), moderate (**4c**), and least active (**4a**) compounds in the active site of *h*CA-II are presented in Figure 6. The docked orientation of all active compounds and the binding interaction of compound **4d** are shown in Figure 7.

## Conclusion

Quinazolinone derivatives (**4a-p**) were synthesized in search of new therapeutic agents for glaucoma and other diseases associated with hyper activity of CA-II. The *in-vitro* results showed that these skeletons displayed moderate to significant inhibition of both the enzymes (*b*CA-II and *h*CA-II). Compounds **4c**, **4e**, **4f**, **4l**, and **4m** showed superior activity against *b*CA-II, while compounds **4g**, **4c**, **4d**, and **4o** were found to be significantly active against *h*CA-II. The structure-activity relationship reflected that the nitro group on phenyl ring at R position plays an important role in the overall inhibitory activities of compounds. Among the tested compounds, **4g** and **4o** are more selective for *h*CA-II. Moreover, kinetics studies showed the competitive behavior of inhibition of this series. Additionally, molecular docking predicted that the compounds bind efficiently with Zinc ion and several residues within the active site, therefore, through appropriate fitting and binding, these compounds effectively inhibit both *b*CA-II and *h*CA-II enzymes.

## Data Availability Statement

The original contributions presented in the study are included in the article/supplementary material, further inquiries can be directed to the corresponding author/s.

## Author Contributions

AA-H and ZS conceived and designed the study. ZK synthesized all compounds. AK and MK performed *in-vitro* assay. MK and SH performed the computational studies and analyzed the data. AK wrote the manuscript with input and comments from MK, SH, ZK, ZS, and AA-H. All authors have read and approved the final version of the manuscript.

## Conflict of Interest

The authors declare that the research was conducted in the absence of any commercial or financial relationships that could be construed as a potential conflict of interest. The handling Editor declared a past co-authorship with one of the authors with the authors AK, SH, and AA-H.
